# Puberty suppression in adolescents with gender dysphoria: an emerging issue with multiple implications

**DOI:** 10.3389/fendo.2024.1309904

**Published:** 2024-06-14

**Authors:** Grigoria Betsi, Panagiota Goulia, Sophia Sandhu, Paraskevi Xekouki

**Affiliations:** ^1^ Endocrinology and Diabetes Clinic, University Hospital of Heraklion, University of Crete School of Medicine, Heraklion, Greece; ^2^ Department of Psychiatry, Cambridgeshire and Peterborough National Health Service (NHS) Foundation Trust, Cambridge, United Kingdom; ^3^ General Practice, Bridge Street Medical Practice, Cambridge, United Kingdom

**Keywords:** puberty suppression, gender dysphoria, transgender, GnRH, transgender and fertility, transgender and bone, transgender and mental health, gender incongruence

## Abstract

Controversy exists over puberty suppression (PS) in adolescents with gender dysphoria (GD). PS is preferentially achieved with GnRH analogues. By preventing the development of secondary sex characteristics, PS may improve psychological functioning, well-being, quality of life, emotional and behavioral (especially internalizing) problems and depressive symptoms, thus decreasing suicidality. PS can also extend the diagnostic period and give transgender adolescents time to explore their gender identity. GnRHa may also decrease the need for feminization/masculinization surgery. However, 2-year treatment with GnRHa may result in bone mass accrual retardation (decrease in BMD/BMAD z-scores), growth velocity deceleration (decrease in height SDS), increase in fat mass, temporary pause in oocyte/sperm maturation. The most common side effects of GnRHa are hot flashes, mood fluctuations, fatigue and headache. They are usually mild and rarely lead to GnRHa discontinuation. Based on current scientific evidence, PS could be recommended to adolescents who meet the diagnostic criteria of gender incongruence (by DSM-5 and/or ICD-11) and have long-lasting intense GD, which aggravates with puberty onset. Before initiating PS, possible mental issues should be addressed and informed consent (by the adolescent/caregiver) should be given, after counseling on probable reproductive effects of GnRHa. GnRHa can only be started after the adolescent has entered Tanner stage 2. Nevertheless, published studies are inadequate in number, small in size, uncontrolled and relatively short-term, so that it is difficult to draw safe conclusions on efficacy and safety of GnRHa. Large long-term randomized controlled trials are needed to expand knowledge on this controversial issue and elucidate the benefit and risks of PS.

## Introduction

Individuals whose gender identity differs from the sex assigned at birth have probably existed since ancient times. Rapid progression on diagnosis and management of gender dysphoria (GD) in adolescents has been made over the last decades, leading to an increase in the number of individuals seeking or referred for endocrine care (1, 2). The first clinic for treatment of transgender youth opened in Amsterdam in 1987 and since then gender centers have proliferated.

Gender dysphoria/incongruence is defined according to ICD-11 and/or DSM-5-TR (**Table 1**) classifications as a marked persistent incongruence between one’s experienced and assigned gender. The discordance of primary and/or developed/anticipated secondary sex characteristics with the expressed gender “often leads to a desire to ‘transition’” in adolescence and adulthood (1, 3). Distress is not a required indicator of the ICD-11 classification of gender incongruence (1).

**Table 1:** Diagnostic criteria for GD.

DSM-5 criteria for GD	ICD-11 criteria for gender incongruence
A. A marked incongruence between one’s experienced/expressed gender and assigned gender of at least 6 months in duration	marked and persistent incongruence between an individual’s experienced gender and the assigned sex
- in adolescents and adults, at least 2 of the following: A marked incongruence between one’s experienced/expressed gender and primary and/or secondary sex characteristics (or in young adolescents, the anticipated secondary sex characteristics) A strong desire to be rid of one’s primary and/or secondary sex characteristics because of a marked incongruence with one’s experienced/expressed gender (or in young adolescents, a desire to prevent the development of the anticipated secondary sex characteristics) A strong desire for the primary and/or secondary sex characteristics of the other gender A strong desire to be of the other gender (or some alternative gender different from one’s designated gender) A strong desire to be treated as the other gender (or some alternative gender different from one’s designated gender) A strong conviction that one has the typical feelings and reactions of the other gender (or some alternative gender different from one’s designated gender)	Adolescence and Adulthood: desire to ‘transition’, in order to live and be accepted as a person of the experienced gender, through hormonal treatment, surgery or other health care services to make the individual’s body align, as much as desired and to the extent possible, with the experienced gender. ∘ The diagnosis cannot be assigned prior the onset of puberty. ∘ Gender variant behaviour and preferences alone are not a basis for assigning the diagnosis
- in children: at least 6 of the following (one of which must be the first criterion): A strong desire to be of the other gender or an insistence that one is the other gender (or some alternative gender different from one’s assigned gender) In boys (assigned gender), a strong preference for cross-dressing or simulating female attire; or in girls (assigned gender), a strong preference for wearing only typical masculine clothing and a strong resistance to the wearing of typical feminine clothing A strong preference for cross-gender roles in make-believe play or fantasy play A strong preference for the toys, games or activities stereotypically used or engaged in by the other gender A strong preference for playmates of the other gender In boys (assigned gender), a strong rejection of typically masculine toys, games, and activities and a strong avoidance of rough-and-tumble play; or in girls (assigned gender), a strong rejection of typically feminine toys, games, and activities A strong dislike of one’s sexual anatomy A strong desire for the physical sex characteristics that match one’s experienced gender	Pre-pubertal childhood: incongruence must have persisted for about 2 years. ∘ a strong desire to be a different gender than the assigned sex; ∘ a strong dislike on the child’s part of his or her sexual anatomy or anticipated secondary sex characteristics and/or ∘ a strong desire for the primary and/or anticipated secondary sex characteristics that match the experienced gender; and ∘ make-believe or fantasy play, toys, games, or activities and playmates that are typical of the experienced gender rather than the assigned sex. Gender variant behaviour and preferences alone are not a basis for assigning the diagnosis.
B. The condition is associated with clinically significant distress or impairment in social, occupational, or other important areas of functioning. ▪ Specify if the condition exists with a disorder of sex development	

Prevalence of GD between 0,6 and 1,7% has been reported in children and adolescents (2) and varies greatly based on definition, country, year, source of data (from health-system records or self-reports) etc., with higher rates reported in recent years and most estimates obtained from survey studies conducted in schools in the United States or Western Europe (1, 2). The etiology of GD is largely unknown; however current research suggests that psychosocial and biological factors play a role in the development of gender identity (2).

Adolescence is accompanied by rapid physical, emotional, cognitive maturation. Body changes, sexual maturation and increased growth rate during puberty may worsen the distress of adolescents with GD. Puberty suppression (PS) has been proposed as a means of preventing unpleasant development of sex characteristics, thus alleviating the distress they frequently cause. However, PS in transgender adolescents is a subject of debate. Scientific controversies exist regarding safety and necessity, while legal and ethical barriers also exist.

## Goal of PS in GD

Goal of early PS in adolescents with GD is to prevent further permanent development of undesirable endogenous secondary sex characteristics, which could lead to substantial distress (3). Such features include Adam’s apple, deep voice, brow and mandible prominence, tall stature and facial hair in those assigned male at birth (AMAB), and breasts, female body shape, and relative short stature in those assigned female at birth (AFAB).

PS can prolong the diagnostic period and offer transgender adolescents the time needed to explore their gender identity (1, 3), before deciding to proceed to partially reversible (testosterone/estrogen) and irreversible (surgical) treatments. During PS the adolescents can think over and verify their decision, discuss hesitations, doubts and fears with their health care professionals (HCP) and parents/guardians. They can also go through social transition and try living like adolescents of the experienced gender. If the adolescents regret treatment, reversible GnRHa can be discontinued and puberty usually recommences a few months later.

## GnRHa mechanism of action and formulations

GnRH agonists (GnRHa) are recommended to suppress puberty (3). At the onset of puberty, GnRH pulsatile secretion by hypothalamus begins. Intermittent GnRH release is required for normal gonadotropin secretion, while continuous GnRH administration results in downregulation of GnRH receptors on anterior pituitary and suppression of gonadotropins and gonadal steroids (after an initial transient increase). Half-life of GnRH is about 3-6 minutes, because it is not bound to serum proteins, thus it is rapidly degraded by proteases. GnRH analogs are less vulnerable to proteolysis, have longer half-life and higher affinity for GnRH receptor due to substitution of amino-acids of natural GnRH, mainly at position 6 (4).

Leuprolide, triptorelin, goserelin, histrelin and nafarelin are available GnRHa that can be administered subcutaneously (or intramuscularly) for PS (5) (**Table 2**).

**Table 2:** Available GnRH analogs for PS.

GnRH analog	Dose
Leuprolide (acetate)	3,75-7,5 mg monthly im or sc 11,25 mg im every 3 months im or sc 22,5 mg sc every 3 months, 30 mg sc every 4 months 45 mg sc every 6 months
triptorelin	3,75 mg every 4 weeks (every 2 weeks during the first month) im or sc 11,25 mg every 3 months 22,5 mg every 6 months
goserelin	3,6 mg every 4 weeks sc 10,8 mg every 3 months sc
histrelin	50 mg (delivering 65 mcg/d), yearly sc (implant surgically inserted in the upper inner arm)
nafarelin	1600 to 1800 μg 4 times per day intranasally (nasal spray)

## Diagnosis of GD in adolescence and criteria for GnRHa treatment

Endocrine society advises that diagnosis of GD/gender incongruence in adolescents should be made only by mental health professionals (MHP) who can use DSM and/or ICD, who are skilled in diagnosis of psychiatric disorders and can distinguish between GD and psychiatric conditions with similar manifestations (e.g. body dysmorphic disorder) and know the criteria for PS and other gender-affirming treatment in adolescents (3). DSM–5 clearly articulates that “gender non-conformity is not in itself a mental disorder”.

World Professional Association for Transgender Health (WPATH) recommends that HCP only recommend gender-affirming treatments requested by transgender adolescents when “the adolescent meets the diagnostic criteria of gender incongruence” (DSM-5, ICD-11) (1). The Endocrine Society suggests that “adolescents who meet diagnostic criteria for GD/gender incongruence, fulfill criteria for treatment, and are requesting treatment should initially undergo treatment to suppress pubertal development” (3).

WPATH and Endocrine Society suggest some eligibility criteria for GnRHa treatment of adolescents (3). It is recommended that a trained MHP confirms diagnosis of (suppressed or expressed) intense persistent GD, which deteriorated with puberty onset. The MHP establishes that possible coexisting mental or physical health or social problems, which might interact with therapy and jeopardize compliance, have been managed, and that the adolescent’s condition and functioning are stable. The adolescent should be informed about the efficacy and possible adverse effects of GnRHa (including potential temporary impact on fertility) and about choices for fertility preservation. Informed consent should be given by the adolescent, provided that he/she has adequate cognitive and emotional maturity and mental capacity (assessed by the MHP). The parent(s)/guardian should give consent before and support during GnRHa, if the adolescent is younger than the age of legal consent. A pediatric endocrinologist (or pediatrician trained in growth assessment) confirms the indication for GnRHa, the absence of contraindications and the first signs of puberty (Tanner stage 2).

## Persistence of childhood GD and recommended time for GnRHa initiation

Gender diversity/incongruence can be expressed in prepubertal children; however, it frequently desists into adolescence, which may be a critical period for gender identity development (1, 3). A study of 139 boys referred at a mean age of 7,49 years (range 3,33–12,99) for assessment of GD, showed that GD persisted in only 12,2% during follow-up (until mean age 20,58 years) (6). Another study of 127 adolescents, referred for GD before the age of 12, showed that persistence of childhood GD in adolescence was more likely in AFAB and older children and in those who had experienced a social role transition (7). The Endocrine Society recommends “against puberty blocking and gender-affirming hormone treatment in prepubertal children with GD/gender incongruence” (3).

Thus, persistence of GD should be evaluated after the first signs of puberty (3), which consist of “the breast bud stage” in girls (elevation of breast and papilla “as small mound” and expansion of areolar diameter) and testes volume greater than 4 ml in boys (accompanied by slight increase in penis length and scrotum size and change in the texture and reddening of scrotum skin). These first changes of puberty typically occur at the age of 9-14 in boys and 7-13 in girls. The discomfort transgender adolescents feel after the first physical changes of puberty can contribute to diagnosis confirmation of GD persistence. Therefore, Endocrine Society and WPATH suggest that HCPs begin PS in eligible transgender adolescents “only after they first exhibit physical changes of puberty (Tanner stages G2/B2)” (1, 3).

Measurement of FSH/LH and estradiol/testosterone (by ultrasensitive assays) in early morning blood samples may supplement clinical examination and confirm hypothalamus-pituitary-gonads (HPG) axis activation at puberty onset (3).

GnRHa administration could probably be initiated in an adolescent at Tanner stage 1 only in cases of constitutional delay in growth and puberty. In these cases GnRHa may be added soon after beginning estrogen or testosterone (1).

GnRHa can be administered at later stages of puberty (Tanner 4 or 5) to deter further breast development in AFAB and facial hair growth or further voice deepening in AMAB, although secondary sex characteristics will not regress completely. GnRHa can also be used in late puberty to suspend erections in AMAB or halt unpleasant menses in AFAB (alternatively, lynestrenol or medroxyprogesterone can be administered to cause amenorrhea) (3).

However, it is uncertain if GnRHa initiation before completion of puberty influences further gender identity development or if it contributes to GD persistence (8). There are concerns that PS may deter adolescents with GD from feeling comfortable with birth-assigned gender. By suppressing gonadal hormones, GnRHa may restrict sexual desire, thus preventing adolescents from having age-appropriate (socio-) sexual experiences, which affect gender identity development.

A retrospective cohort study of 434 transgender adolescents (71,9% AFAB) of mean age 15,4 years (at first visit) showed that GnRHa did not increase the likelihood of subsequent gender-affirming hormone therapy (GAHT) use. GnRHa treatment was associated with longer time between the first visit and GAHT initiation. In multivariate analysis GnRHa use was independently associated with lower risk of GAHT initiation. These associations still existed among 54 adolescents 10 to 13 years old (at first visit), suggesting that GnRHa could be offered to young transgender adolescents without worry for affecting their decision to proceed to GAHT treatment. Adolescents between 14 and 17 years old at first visit received GnRHa less frequently, but were more likely to begin GAHT and after a shorter time, as compared to those between 10 and 13 years old (9).

## Effectiveness of GnRHa in gonadal and puberty suppression

Preliminary results of the first 21 adolescents, treated (according to the Dutch protocol) with triptorelin (3,75 mg monthly) for at least 2 years, showed sufficient suppression of gonadotrophins and gonadal steroids (estradiol in AFAB, testosterone in AMAB) to prepubertal levels. Puberty did not develop. Testicular volume reduced in AMAB (10).

GnRHa are effective in HPG axis suppression in transgender adolescents, similarly to central precocious puberty (CPP). A retrospective review of medical records of 60 youth (30 transgender, 30 CPP) showed that reduction in FSH/LH and testosterone/estradiol was not significantly different during GnRHa treatment between transgender and CPP youth. FSH levels were lower after histrelin treatment of transgender adolescents compared with leuprolide treatment (11).

The efficacy of GnRHa in PS was also shown in a prospective study of 116 (57,8% AFAB) transgender adolescents. Gonadotropins and estradiol were suppressed within 3 months of triptorelin. Testosterone levels were also suppressed in AMAB adolescents. No adjustment in GnRHa treatment was necessary in anyone due to insufficient suppression. GnRHa resulted in testicular volume reduction in 88% (43 of 49) AMAB, in 33 of them it decreased from 13,9 at baseline to 8,6 mL at 12 months. Among four AFAB with breast Tanner stage 2 at presentation, one manifested complete regression of breast development after 6 months of GnRHa, while menses ceased in those who had had menarche usually after a withdrawal bleed (12).

In a prospective observational study in the UK of triptorelin monotherapy, gonadotropins were suppressed by 6 months and remained suppressed thereafter in all 44 transgender adolescents studied. Most AFAB were at stage 4 and post-menarcheal (at triptorelin initiation) and secondary amenorrhea occurred in all AFAB during the first 3 months of GnRHa (13).

Another prospective study of 36 transgender adolescents in Italy confirmed reduction in gonadotropins by GnRHa. FSH in AMAB and LH in AFAB decreased nonlinearly and more rapidly during the first months of triptorelin. Testosterone declined in AMAB in the first half of triptorelin treatment and stabilized afterwards. Estradiol levels fell significantly and approximately linearly in AFAB in the first 10 months of triptorelin. Statistically significant transition to earlier Tanner stage was reported after GnRH in 9 patients (25%). Menstruation ceased at T3 in all 20 AFAB who had menarche. Hair growth and acne severity receded in all (especially AMAB) during triptorelin (14).

In order to evaluate adequate HPG axis suppression, Endocrine Society suggests measurement of FSH/LH and estradiol/testosterone at baseline and every 6-12 months during PS. If there are clinical (menses, erections, hair growth) and/or laboratory evidence of insufficient HPG suppression, GnRHa dose can be raised or the interval between the doses can be shortened (3).

## Mental health in adolescents with GD

Development of primary and/or secondary sex characteristics during puberty can cause adolescents with GD serious psychological distress (3). Distress can hinder activities of daily living, resulting in depression and suicidal ideation.

The role of GD as a stressor could be explained by the minority stress model, which highlights the “excess exposure to social stress faced by sexual minority populations due to their stigmatized social status” (15). Many children have to confront social stigmatization and bullying at school. Nahata et al. reported that 58,2% of 79 young individuals with GD had documented school victimization (16).

Many studies have pointed out high rates of mental health concerns in untreated transgender adolescents. In a retrospective medical record review of 79 young individuals with GD, 78,5% were diagnosed with depression, 63,3% with anxiety, 74,7% reported suicidal ideation, 55,7% exhibited self-harm and 30,4% made suicide attempt(s) (15). Tordoff et al. noted that 56,7% of 104 transgender and nonbinary youth had moderate to severe depression, 50% had moderate to severe anxiety and 43,3% reported self-harm or suicidal thoughts before receiving gender-affirming treatment (17).

However, de Vries et al. suggested that the majority (67,6%) of adolescents with GD don’t have concurrent psychiatric disorders. Anxiety occurred in 21%, mood disorders in 12,4% and disruptive disorders in 11,4% of the adolescents (18).

Many adolescents with GD are referred for treatment due to poor psychological functioning. In a cross-sectional study in Amsterdam, 272 adolescents referred to a specialized gender identity clinic manifested more behavioral and emotional (especially internalizing) problems, more self-harm/suicidality, and poorer peer relations before gender-affirming treatment compared with cisgender controls from the general population (19).

Therefore, HCPs should “undertake a comprehensive biopsychosocial assessment of adolescents” who present with GD and request “transition-related care”, which is recommended only when “the adolescent’s mental health concerns (if any) that may interfere with diagnostic clarity, capacity to consent, and/or gender-affirming medical treatments have been addressed”. Moreover, “it is critical to differentiate gender incongruence from specific mental health presentations, such as obsessions and compulsions,…, broader identity problems,…”. Treatment of transgender youth in multidisciplinary clinics in close cooperation with MHPs is beneficial (1).

Haltering the stress that adolescents may experience during puberty by hormonal suppression and allowing them some time to explore their gender identity can provide some relief to the distress from the development of secondary characteristics (**Table 3**).

**Table 3:** Effect of PS on mental health.

	Positive effect of PS on mental health	Negative/neutral effect of PS on mental health
Chen D et al. 2023 (20)	• Higher scores for appearance congruence, positive affect and life satisfaction, • lower scores for depression and anxiety (in AMAB who started GnRHa at early puberty)	
Costa R et al. 2015 (21)	• better psychosocial functioning after 1 year PS (compared with psychological support only)	
De Vries A et al. 2011 (22)	• Improvement in general functioning, • reduction in depressive symptoms, • reduction in behavioral and emotional problems and in internalizing and externalizing behavior T-scores	No significant change in anxiety, anger, body satisfaction and GD
De Vries A 2014 et al. (23)	• Steady improvement in psychological functioning (especially in AFAB), decrease in depression, objective and subjective well-being • decrease (not statistically significant) in anxiety only in AFAB (during and longer after PS)	Persistence in GD and body image difficulties AFAB reported more dissatisfaction with secondary sex characteristics after PS, (GD resolved after subsequent of GAHT and gender-reassignment surgery)
Tordoff D et al. 2022 (17)	• 60% lower odds of moderate to severe depressive symptoms during 1^st^ year of PS or GAHT	no association between PS or GAHT and moderate to severe anxiety
Van der Miesen A et al. (19)	• lower frequency of self-harm/suicidality (than clinic-referred GD) less emotional and behavioural problems, with lower score on internalizing (emotional) problems • lower score on peer relations (than clinic-referred GD)	higher score on peer relation problems (than cisgender controls from general population)
Khatchadourian K et al. 2014 (24)	• reduction in suicide attempts (from 12% to 5%)	
Turban J et al. 2020 (25)	• lower odds of lifetime suicidal ideation	
Becker-Hebly I et al. 2021 (26)	• improved (“within the norm mean”) mental and physical health-related quality of life (lower than age-matched controls, statistical significance not tested)	Persistence in internalizing problems during 2 years follow-up after GnRHa
Kuper L et al. 2020 (27)	• Improvement in body dissatisfaction during gender-affirming treatment (including 10 adolescents receiving PS only)	No decrease in social anxiety symptoms
Carmichael P et al. 2021 (13)	• Experience/satisfaction: positive (or mixed) changes for the majority (e.g. feeling happier, better relationships with family and peers) • Mood changes at 6–15 months: improved mood (49%), mixed changes (feeling happier but with some mood swings; 15%) • predominantly positive or neutral change in family (feeling closer/accepted) and peer (feeling more sociable or confident, widening circle) relationships • At 6–15 months, 66% reported positive changes in gender role (feeling more feminine/masculine, living in preferred gender identity in more/all areas of life, feeling more secure in gender identity), no negative change	no significant change in psychological functioning and in self-harm during 3 years of triptorelin (started at late puberty for the majority) 24% reported negative mood changes at 6-15 mths
Fisher A et al. 2023 (14)	• significant reduction in externalizing and internalizing problems (lower scores on scales related to thought and social problems, somatic complaints, and anxious–depressive symptomatology) • improvement in body uneasiness lower repulsion by life component of suicide tendency scale less depressive symptoms (lower self-rating scale in Beck Depression Inventory) • lower levels of anxiety (lower self-rating scale in Beck Anxiety Inventory)	no significant improvement in aggressive behavior, rule-breaking behavior and attention problems

In Trans Youth Care–United States (TYCUS), a prospective, observational study of 315 transgender and nonbinary youth (mean age 16), participants who had not experienced considerable endogenous puberty changes had better psychosocial functioning. Twenty adolescents who started GnRHa at Tanner stage 2-3 and 4 adolescents who initiated GAHT without previous GnRHa treatment at stage 3 (due to relatively late onset of puberty) had greater positive affect and less anxiety before starting GAHT than those who had started GAHT in later puberty (thus experiencing gender-incongruent puberty). These differences were statistically significant only among 20 early-pubertal AMAB (20).

Transgender children (aged 3–12 years) with early social transition don’t manifest increased levels of depression, but have slightly increased levels of anxiety, with their rates of internalizing problems being considerably lower than children with GD living as their birth-assigned gender (28). Access to gender-affirming therapy may help social transition, potentially reducing the rates of distress and school victimization.

Individuals who received PS have been found to have better psychological outcomes, improved well-being and higher quality of life. Adolescents with GD had significantly better psychosocial functioning after one year of GnRHa treatment than those who received psychosocial support alone (21). In a Dutch longitudinal observational cohort study of 70 adolescents, general functioning improved during PS, so that no one discontinued GnRHa and all proceeded to GAHT (22).

The first longer term study of 55 young transgender adults, who had received PS during adolescence, showed that psychological functioning steadily improved (especially in transmen), objective and subjective well-being (post-treatment) was comparable to peers in general population and none reported regret during PS (23).

However, Carmichael et al. found no significant change in psychological functioning during 3 years of triptorelin treatment (started at late puberty for the majority) of 44 adolescents with GD, although the majority reported positive changes (e.g. feeling happier, better relationships with family and peers) (13).

Depression has been shown to decrease during PS. De Vries et al. and Fisher et al. found a reduction in depressive symptoms in adolescents with GD during GnRHa treatment (14, 22, 23). TYCUS study showed less depression in transgender youth, whose puberty was suppressed or started later (20). In a prospective observational cohort study, treatment of 104 youth with puberty blockers or GAHT was associated with 60% lower odds of moderate to severe depressive symptoms during the first year, while significant increase in moderate to severe depression was noticed in youth who had not received treatment (17).

PS may decrease suicidality. Tordoff et al. showed that PS or GAHT was associated with 73% lower odds of self-harm or suicidal thoughts in youth (17). Van der Miesen et al. found that among 178 transgender on puberty blockers, self-harm/suicidality was less frequent than in clinic-referred transgender and similar with non-clinical peers (19). Khatchadourian et al. noted a reduction in suicide attempts (from 12% to 5%) after GnRHa treatment of 27 youth with GD in Canada (24). A cross-sectional survey of 20619 transgender adults (aged 18 to 36 years) showed that those (n=89) who had received PS during adolescence had lower odds of lifetime suicidal ideation (in comparison with those who wanted, but did not receive PS) (25). Fisher et al. found lower repulsion by life leading to lower suicidal tendency during triptorelin treatment, which was associated with the decrease in FSH/LH, in waist circumference and in acne severity in AMAB (14). However, Carmichael et al. found no significant change in self-harm during triptorelin treatment (13).

Emotional and behavioral, especially internalizing, problems may improve after GnRHa treatment. In the study by van der Miesen et al., transgender adolescents receiving PS had less emotional and behavioral problems, as they scored lower on internalizing (emotional) problems, in comparison to cisgender peers from the general population as well as to transgender clinic-referred adolescents. Regarding peer relations, transgender adolescents on PS scored less than referred transgender but higher than cisgender adolescent controls (19). De Vries et al. noticed decreased behavioral and emotional problems and lower internalizing and externalizing behavior t-scores during PS (22, 23). Fisher et al. found that externalizing and internalizing problems decreased significantly after GnRHa treatment of trans-adolescents (14). However, in a small German cohort study of 11 adolescents with GD (73% AFAB), internalizing problems persisted during 2 years (average) follow-up after GnRHa treatment (26).

Nevertheless, the latter study showed that GnRHa treatment resulted in improved (“within the norm mean”) mental and physical health-related quality of life, which was lower than age-matched controls pretreatment, although statistical significance was not tested (26).

Anxiety may not decrease significantly during PS. Tordoff et al. found no association between PS or GAHT treatment of youth with GD and moderate to severe anxiety (17). Kuper et al. found that social anxiety symptoms did not decrease during PS (27). De Vries et al. (2011) reported that anxiety and anger did not change during GnRHa treatment of 70 adolescents with GD (22), although the longer term study by de Vries et al. (2014) noted a decrease (not statistically significant) in anxiety only in transmen during and later after PS (23). Moreover, Fisher et al. found a reduction in anxiety during triptorelin treatment of 36 transgender adolescents, which was associated with the decrease in FSH (14).

PS may not amend body dissatisfaction. De Vries et al. (2011) noticed that body satisfaction and GD did not change significantly during GnRHa treatment of 70 adolescents (22). The longer-term study by de Vries et al. (2014) showed that GD and body image difficulties persisted during PS (with transmen reporting more dissatisfaction with secondary sex characteristics after PS), although GD resolved after subsequent administration of GAHT and gender-reassignment surgery (23).

However, other studies have showed that body changes during PS may have a positive psychological effect on trans-adolescents. In TYCUS study, transgender youth who had not gone through significant physical changes of endogenous puberty had greater appearance congruence, which was associated with better psychosocial functioning, although less improvement in appearance congruence was noticed after two years of gender-affirming treatment in youth treated since early puberty than those starting GAHT in later puberty (20). Kuper et al. reported that body dissatisfaction of youth improved during gender-affirming treatment (including 10 adolescents who received PS only, starting early at mean age of 13,7 years) (27). Fisher et al. concluded that gender-affirming physical changes (e.g. reduction in WHR and acne severity in AMAB) induced by triptorelin treatment of 36 transgender adolescents could account for improvement in body uneasiness and in psychological functioning (14).

The overall positive impact of PS on mental health highlights the need for worldwide availability of gender-affirmative care for transgender adolescents to help alleviate possible psychological problems, reducing them to rates indistinguishable from the general population.

However, there is a limited number of studies focusing on the psychological effects of PS on adolescents with GD (**Table 3**), which highlights the importance of the need for further prospective studies in this field. Furthermore, the small number of participants in the existing studies, the variety in studied outcomes, the use of different tools and the different time periods between initial assessments and follow-up prevents us from drawing firm conclusions.

A matter of discussion is the ethical and legal issues around medical treatment of children with GD. Judicial Review for ‘Bell V’s Tavistock Case’ in the UK ruled that children younger than 16 years are incapable of giving informed consent to medical interventions for GD; however, the Court of Appeal subsequently reversed High Court’s decision, concluding that determination of a child’s competence to consent should be decided on an individual basis by clinicians and parents (29). However, such judgements cannot be generalized, since laws vary across the globe.

## GD persistence into adulthood and high rate of post-GnRHa GAHT use

A recent cohort study of 720 transgender (69% AFAB) individuals, who began GnRHa treatment in adolescence (median age at GnRHa start 14-16 years) for a minimum duration of 3 months (before adding GAHT) showed that 98% of the study population continued gender-affirming hormones at follow-up into adulthood (30).

In a retrospective study in Canada, transition to testosterone or estrogen was made for 14 of 15 AFAB and for 5 of 11 AMAB (respectively) receiving GnRHa, which had been started at Tanner stage 4-5 for the majority (59%) (24).

A retrospective study in a gender clinic in the Netherlands showed that the majority of adolescents who had started GnRHa subsequently continued treatment with GAHT and only a minority discontinued GnRHa. Among 143 adolescents (73,4% AFAB) who began GnRHa treatment (at a median age of 15-16), 87% started GAHT after a median duration of 0,8 years on GnRHa. Nine (6%) adolescents (8 of whom AFAB) stopped GnRHa after a median of 0,8 years, five of whom no longer wanted gender-affirming treatment, while three adolescents discontinued due to possible side effects (31).

## GnRHa administration may reduce the necessary concurrent doses of GAHT

A retrospective review of outpatient medical records of 83 transgender adolescents (73% AFAB), 17 of whom received GnRHa (median age at GnRHa start 14,5 years for AMAB, 13,9 for AFAB), showed that initiation of GnRHa before GAHT was associated with significantly lower average dose of oral estradiol/subcutaneous testosterone. Frequency of adverse effects of GAHT was not significantly different between those taking and those not taking GnRHa (32).

## GnRHa may reduce the need for feminization/masculinization surgery

Transgender individuals who had received PS at early stages may not need to undergo most of the non-genital surgeries. In a retrospective study of youth with GD in Canada one (out of 15) AFAB did not need chest surgery, because GnRHa was started early and prevented breast growth (24). Mastectomy with chest reconstruction (and liposuction) in AFAB, facial feminization surgery and chondrolaryngoplasty in AMAB may be unnecessary, because development of biological sex characteristics has been impeded. Penile inversion vaginoplasty may be impracticable in AMAB who had received PS early, because GnRHa may result in penoscrotal hypoplasia (leaving inadequate amount of penile tissue), in these cases intestinal vaginoplasty is more appropriate (33).

## Alternatives to GnRHa in late-pubertal transgender adolescents

Proandrogenic and antiandrogenic progestins are a cheap oral alternative in late-pubertal AFAB and AMAB, respectively, who have already developed secondary sex characteristics.

Lynestrenol is an androgenic progestin, converted to norethisterone, which reduces LH, SHBG, total testosterone, while free testosterone may rise slightly (33). In a retrospective analysis of data from 45 AFAB adolescents at Tanner stage B4 or further treated with lynestrenol monotherapy (and in combination with testosterone for at least 6 months) showed than mean LH and E2 (not FSH) levels decreased during the first 6 months and remained stable in the next 6 months of lynestrenol monotherapy, while LH and FSH were fully suppressed after combination therapy. SHBG and total testosterone fell significantly, although free testosterone increased non-significantly in the first 6 months of lynestrenol and remained unchanged in the next 6 months. The most common side effects of treatment were headaches, hot flushes, and fatigue. Hematocrit rose significantly in the first 6 months of monotherapy and combination but remained stable thereafter. Acne increased non-significantly during lynestrenol monotherapy and appeared more frequently during the first 6 months of combination. Metrorrhagia was mainly reported in the first 6 months, but was significantly reduced in the following 6 months of monotherapy and increased slightly during combination. Weight and BMI significantly increased in the first 6 months and returned to baseline after 12 months of lynestrenol monotherapy, while significant weight gain was noticed after combination. Triglyceride levels did not change, although mean HDL fell and mean LDL increased significantly in the first 6 months of lynestrenol, the latter did not change during combination. Glucose levels, HbA1c and HOMA were not significantly altered during treatment (34).

Cyproterone acetate (CA) has been used in late-pubertal AMAB due to its antiandrogenic effects, resulting mainly through competitive inhibition of binding to androgen receptor. According to a retrospective analysis of data from CA treatment of 27 AMAB (presenting at Tanner stage G4), more than half of the studied youth reported decreased facial hair growth (reduced shaving frequency) and some reported less spontaneous erections. Breasts developed during CA monotherapy (29,6% to Tanner B2-B3) and further (66,7% to Tanner B3, 9,5% reached Tanner B4) during subsequent combination treatment (CA with estrogen), although breast size was small in most cases. Fatigue was the most common side effect, reported in 37% of AMAB during CA monotherapy, receding in the majority after addition of estrogens. Other relatively common side effects of CA (monotherapy) include emotionality (11,1%) and breast tenderness (7,4%). Growth was significantly less compared with age-matched peers, because all adolescents had already reached at least Tanner G4. Weight gain during CA monotherapy was small, although it was greater than in age-matched male peers, but rise in height was also more than in controls, so that BMI was not significantly altered. CA did not have a significant effect on glucose levels, HbA1c, HOMA index and LDL, while triglycerides declined. FSH levels were slightly reduced, but not suppressed, during the first 6 months of CA. CA resulted in a significant progressive reduction in testosterone levels (below male, but not within female, reference interval). Prolactin increased during CA, but none of the treated AMAB manifested galactorrhea. Neither increase in PRL nor decrease in testosterone were associated with breast development (35).

CA and lynestrenol treatment for approximately one year can induce body composition changes in line with the desired gender appearance. However, CA may restrain pubertal bone mass accrual (mainly at LS), while lynestrenol has probably no significant impact on normal bone development. In a prospective study of 21 AMAB and 44 AFAB at Tanner stage G4 or G5 treated with CA or lynestrenol (respectively) for 10,6 months (mean), mean total testosterone and estradiol reduced, FSH and LH decreased only during lynestrenol, free testosterone and total testosterone-to-estradiol ratio decreased during CA and SHBG declined (raising free testosterone) during lynestrenol. PINP reduced significantly during lynestrenol and CA, reduction was greater (by 46,5%) during CA, which also induced a s-CTX drop by 17,1%. During lynestrenol, lean mass increased significantly, resulting in reduced body fat percentage (compared with age-matched female peers), weight and waist/hip ratio (WHR) increased, muscle (not fat) area at left lower leg and at nondominant forearm and grip strength also increased. Thus, lynestrenol induced a more masculine body composition and musculature. During CA, fat mass significantly increased, lean mass decreased, muscle area decreased significantly, while grip strength was not significantly altered, leading to decreased z-scores (compared to male peers). Total hip aBMD (absolute values and z-scores) increased in AFAB and decreased in AMAB. In AFAB femoral neck (FN) and lumbar spine (LS) aBMD absolute values increased significantly, without significant change in z-scores, indicating bone development similar to female peers. However, FN and LS aBMD z-scores decreased during CA (with stable aBMD absolute values). Trabecular volumetric BMD at radius increased in AFAB (similarly as in age-matched control female), while it decreased in AMAB. Periosteal circumferences z-scores of radius and tibia reduced during CA, indicating significantly less periosteal expansion (36).

A cumulative dose-dependent association has been found between CA use and meningioma. CA is a progestogen and meningiomas express progesterone receptors. EMA recommended (in 2020) restrictions in CA use of daily doses of 10 mg or more, only for androgen-dependent conditions, when lower doses or other treatments have failed (37).

Spironolactone can promote feminization, because it is a moderate androgen receptor antagonist, which also partially inhibits 17α-hydroxylase/17,20-lyase, reducing thus androgen synthesis, while it also has a weak estrogen receptor agonist (in the absence of endogenous estrogen) as well as a partial progesterone receptor agonist effect (37). Spironolactone has been used as an antiandrogen in gender-diverse adolescents (38).

In a retrospective study of 330 transwomen, those requesting breast augmentation had used spironolactone more frequently in comparison with other antiandrogens (CA, finasteride, dutasteride). The most common adverse effect of antiandrogens was depression, which was more frequent than with GnRH analog. CA was significantly correlated with depression (8,3%) (39).

Bicalutamide has been tested in a few AMAB as a second-line puberty blocker (alternatively to GnRHa). Bicalutamide antagonizes the androgen receptor, resulting in increased testosterone, which is aromatized to estrogen. A retrospective study of 23 transgender female adolescents (mean age 16) received bicalutamide (50 mg daily), six individuals received estrogen concomitantly. Breast development (≥ Tanner stage III) was noticed within 6 months in 84,6% of the study population, acne and frequency of shaving decreased. Estradiol levels were above 20 pg/dl (except in one subject) (40).

Medroxyprogesterone inhibits HPG axis and it has been used as a contraceptive. A retrospective study of 14 adolescents with GD supported the efficacy of medroxyprogesterone for menstrual cessation in AFABs and for puberty delay in AMABs. None stopped treatment due to adverse effects, three discontinued in order to return to assigned gender (due to psychosocial reasons). Seven AMAB adolescents, presenting at puberty stages 2-4, were treated with oral medroxyprogesterone (4 of whom for 3 years), with initial dose 10–30 mg per os bid and increasing doses until age 15-16 (thereafter dose decreased, while estradiol was added). One AMAB stopped treatment after 6 months. Complete suppression of testosterone was not always achieved with medroxyprogesterone, greater suppression was feasible when starting treatment in early puberty. Six postmenarchal AFAB (Tanner 5) received depot intramuscular medroxyprogesterone (initial dose 150 mg every 3 months, then every 2 months), to stop menses. One AFAB presenting at Tanner 2a received per os medroxyprogesterone (20 mg bid), with subsequent regression of breast tissue (41).

## Effect of PS on bone metabolism

Growth spurt, time of the most rapid height velocity, occurs between Tanner stage 2 and 3 in girls and between stages 3 and 4 in boys. During puberty, bone mass and mineralization increase rapidly. Peak bone mass, which is achieved in early adulthood, reflects bone strength and predicts later development of osteoporosis. Gonadal steroids (estrogen and androgen) and growth-hormone/IGF1, which increase significantly during puberty, have a significant impact on skeletal development. Androgen increase periosteal apposition, expanding bone size and strength in male. Estrogen decrease periosteal apposition and endocortical resorption, so that a calcium reservoir is made for pregnancy and lactation. Estrogen inhibits chondrocyte proliferation at growth plate, leading to epiphyseal fusion and cessation of linear growth (42).

Limitations exist in the normal values used as reference for interpretation of bone density measurements in transgender individuals, because PS results in gonadal hormone reduction, which may differentiate the pattern of bone growth from that expected for the birth-assigned gender. According to International Society for Clinical Densitometry (ISCD) 2019 official positions, “z-scores should be calculated using the normative database that matches the gender identity” of transgender individuals. Because transgender female have lower BMD than cisgender male (before GnRH and GAHT treatments), female reference database in transgender female may be more reliable to evaluate z-score, although it may lead to its overestimation, missing cases of low BMD (for age) and ignoring the need to search for secondary causes of osteoporosis. Male reference database may be appropriate for estimation of z-score in transgender men, because their BMD is close to that of cisgender men. Construction of a reference database for bone density of transgender may be an aim for future research (43).

The Trans Youth Care Study showed high prevalence of low BMD before (or no more than 2 months after) GnRHa initiation among 63 transgender adolescents in early puberty. Low (lower than -2) areal or volumetric BMD z-score was found in 30% of AMAB and 13% of AFAB. These rates are significantly higher than those expected (2,3%) in a normal distribution. AFAB had higher mean BMD z-scores than AMAB, with statistically significant difference at the hip. A negative association was found between age at puberty blocker placement and total hip BMD z-scores, explained (at least in part) by the later puberty onset in males. AFAB and transgender youth with normal BMD reported statistically significantly higher physical-activity scores than AMAB and youth with low BMD, respectively (44).

There is concern that puberty blockers may delay normal bone development and affect peak bone mass (**Table 4**) by inhibiting normal endogenous production of gonadal steroids.

**Table 4:** Effect of GnRHa treatment on bone density.

	BMD/BMAD LS		BMD/BMAD hip		BMD/BMAD LS z-score		BMD/BMAD hip z-score	
	AFAB	AMAB	AFAB	AMAB	AFAB	AMAB	AFAB	AMAB
Klink et al. 2015 (45)*	↓*	↔ *	↓ (FN) *	↔ (FN) *	↓ *	↔ *(decrease, but not significant)	↓ (FN) *	↔ (FN) * (decrease, but not significant)
Navabi et al. 2021 (46)	ND	ND	ND	ND	↓ (+LS BMAD z-score↓)	↓	↓ (TH)	↓ (TH)
Vlot et al. 2017 (47)	↓ (BA≥14)** ↔ (BA<14)**	↔**	↓ (BA≥14)** ↔ (BA<14)**	↔**	↓**	↓ (BA<15)** ↔ (BA≥15)**	↓ (BA≥14)** ↔ (BA<14)**	↔**
Schagen et al. 2020 (48)	↓ (Tanner 4-5) ↔ (Tanner 2-3)	↔	↓	↓ (Tanner 4-5) ↔ (Tanner 2-3)	↓	↓	↓	↓ (Tanner 4-5) ↔ (Tanner 2-3)
Joseph et al. 2019 (49)	↔	↔	↔	↔	↓ ¶ (BMD+BMAD)	↓ ¶ (BMD+BMAD)	↓	↓
Carmichael et al. 2021 (13)	↔*	↔*	↔*	↔*	↓* (12-24 mths) ↔* (36 mths)	↓* (12-24 mths) ↔* (36 mths)	↓* (12-24 mths) ↔* (36 mths)	↓* (12-24 mths) ↔* (36 mths)
Stoffers et al. 2019 (50)	↓	ND	↓	ND	↓	ND	↓	ND

↔ no statistically significant change during GnRHa treatment; BA, bone age; FN, Femoral Neck; ND, No data / Not determined; TH, Total Hip.

*BMD, ** BMAD.

¶ greater decrease after 1 year GnRHa, lower decrease during the 2nd year.

assigned female at birth (AFAB), assigned male at birth (AMAB).

↓, decrease.

Most studies showed that rate of bone development during 2-year GnRHa treatment of transgender adolescents is lower than in age-matched peers. Initial results of 21 adolescents treated with triptorelin for at least 2 years (according to the Dutch protocol) showed that bone density (in LS, non-dominant hip and total body) did not change significantly, while z-scores decreased significantly (10).

Similarly, a prospective observational study of triptorelin monotherapy in 44 adolescents with GD showed that bone development was retarded, with BMD stable at the hip and increased at the LS (after 2 years), but at a slower pace than in age-matched peers. At baseline, most AMAB were at Tanner stage 3 and most AFAB were at stage 4 and post-menarcheal, while no participant was at stage 2. Hip BMD did not change at 12 and 24 months. LS BMD did not change significantly at 12 months, but it increased after 24 months of GnRHa (compared to baseline). LS BMC was greater than baseline at 12 and 24 months. LS and hip BMD z-scores decreased after 12 and 24 months of GnRHa (13).

Another study in the Netherlands analyzed BMD development in 34 transgender after triptorelin monotherapy (for median 1,3-1,5 years), which was initiated at late puberty in the majority, followed by GAHT with continuation of GnRHa until gonadectomy. In AMAB, BMD did not change, while z-score was reduced, but not significantly, during GnRHa monotherapy. In AFAB both LS and FN absolute BMD and z-scores decreased significantly during GnRHa monotherapy (45).

A decrease in BMD z-scores after one year of leuprolide monotherapy was also found in a retrospective study of 116 transgender youth (69% AFAB) in Canada. At baseline more than 80% were at Tanner stage 4-5 and aBMD values were lower in AMAB than in AFAB. LS and left total hip aBMD z-scores decreased significantly after GnRHa treatment. LS bone mineral apparent density (BMAD) z-scores fell significantly among AFAB (46).

A retrospective cohort study of 70 transgender (60% AFAB) adolescents showed decreased BMAD z-scores mainly in the LS (except in “old” AMAB) without significant alteration in BMAD absolute values during GnRHa treatment. During GnRHa, (LS and hip) BMAD and hip BMAD z-score decreased only in “old” [bone age (BA) 14 years or more] AFAB, while LS BMAD z-score decreased in AFAB and in “young” (BA < 15) AMAB adolescents (47).

A significant decrease in z-scores during GnRHa was also evident in an observational prospective study of 29 early-pubertal (Tanner 2-3 at GnRHa start) and 92 late-pubertal (Tanner 4-5) transgender adolescents. During 2 years of GnRHa treatment, LS and hip BMD and BMAD z-scores decreased significantly (except for hip BMAD z-score of early-pubertal AMAB, whose decline was not statistically significant). BMAD was not significantly altered in the LS of AMAB and early-pubertal AFAB and in the hip of early-pubertal AMAB, while a small, but statistically significant, decrease was found in hip BMAD of AFAB and of late-pubertal AMAB and in LS BMAD of late-pubertal AFAB after 2-years GnRHa treatment (48).

A decrease in BMD z-scores during GnRHa, rapid during the first year, was also found in a retrospective review of 70 adolescents with GD. Most AFAB (94,9%) were in mid-late puberty and had menarche, while most AMAB (57%) were early-pubertal (G2-G3). Z-scores were lower in AFAB than AMAB. Z -scores of hip and LS BMD and of LS BMAD decreased significantly after the first year of GnRHa, a lower drop was noticed in hip and LS BMD and LS BMAD z-scores after the second year. However, no significant change was noticed in absolute values of hip or LS BMD or LS BMAD during GnRHa (49).

Another retrospective study of 62 AFAB adolescents showed that LS and hip BMD and BMD z-scores after at least 6 (median 8) months GnRHa monotherapy (at testosterone initiation) were lower than at GnRHa initiation (50).

Reduced bone turnover probably accounts for the decrease in z-scores during PS. Vlot et al. found that levels of bone formation and resorption markers (especially in younger transgender adolescents) reduced during GnRHa treatment in accordance with decreased BMAD z-scores. P1NP (bone formation marker) decreased significantly in young transgender adolescents during triptorelin treatment, a smaller reduction was also found in old AMAB. Osteocalcin was not affected by GnRHa in most adolescents, except an increase found in old AFAB. A decrease of ICTP (resorption marker) was noticed in all groups except old AFAB (47). In the study by Schagen et al. markers of bone formation (P1NP, P3NP and osteocalcin) and of bone resorption (1CTP) fell significantly in AMAB and in early-pubertal AFAB adolescents after 2 years of GnRHa, especially within the first year (during which BMD was stable). In late-pubertal AFAB, P3NP and 1CTP decreased less but significantly during GnRHa (48).

BMD z-scores probably stabilize relatively and do not decrease further after the second year of GnRHa treatment. Carmichael et al. found that LS and hip BMD z-scores (and absolute hip BMD) did not decrease after 36 months of triptorelin monotherapy of 44 transgender adolescents (although LS BMC increased) (13). Schagen et al. showed that aBMD did not change significantly in a few (4 AFAB, 11 AMAB) adolescents treated with GnRHa for 3-4 years (started at mean age 12,6-12,7 years). No further reduction in aBMD z-scores was found in most subjects during the third or fourth year of triptorelin. However, BMD z-scores were lower at 36 months (than before GnRHa start) in the hip of AMAB and in the LS of AFAB (although the decrease was noticed mainly during the first year) (48).

BMD increases during GAHT (following GnRHa) treatment. Delemarre-van de Waal et al. found that hip and LS BMD z-scores increased during GAHT treatment (administered after triptorelin) of 21 transgender adolescents (10). Stoffers et al. noticed that BMD increased during testosterone (especially during the first 6 months) treatment of 62 AFAB adolescents (50). In the study by Klink et al. GAHT treatment (combined with GnRHa) after triptorelin monotherapy increased absolute LS and FN aBMD. LS aBMD z-score improved (not statistically significantly) in AFAB, while it did not increase in AMAB during GAHT (45). In the study by Vlot et al. hip BMAD and z-scores increased in AFAB and did not change in AMAB adolescents after 24 months of GAHT treatment (added to triptorelin at age 16), while LS BMAD absolute values and z-scores increased in all groups (47).

However, increase in BMD during GAHT may not compensate for the decrease in z-scores during PS, especially in AMAB. Vlot et al. observed that z-scores did not return to pre-GnRHa levels in most transgender adolescents after 2-year GAHT treatment (47). Stoffers et al. found that, after 1-2 years of testosterone treatment, LS and left hip BMD and BMAD were not significantly different than at GnRHa initiation, while BMD and LS BMAD z-scores remained lower than before GnRHa (50). Klink et al. showed significantly lower LS BMD z-score (for birth-assigned gender) at age 22 than at GnRHa initiation in AMAB and a trend for decrease in AFAB. At age 22 years, 6 AMAB (40%) had a LS BMD z-score lower than -2. Duration of GnRHa monotherapy was not correlated with BMD and z-scores at age 22 (45). Van der Loos et al. found that BMD z-scores in transgender individuals who had received PS caught up with pre-GnRHa levels after long-term (around 11 years) of GAHT, except for LS in AMAB (where z-scores remained lower than pretreatment values) (51). Thus, concerns are raised about possible delay or attenuation in acquisition of peak bone mass by GnRH treatment of adolescents.

Apart from bone density, GnRHa treatment of transgender adolescents during early puberty may also affect bone geometry development. A retrospective cohort study in Amsterdam of 322 transgender adolescents (67% AFAB, 86% of whom were in late puberty at study entry) showed that the alterations in hip subperiosteal width and endocortical diameter were similar with the reference curve of the experienced gender in study subjects who began GnRHa treatment in early puberty, while they remained within the reference curve for birth-assigned gender in adolescents who initiated GnRHa in mid- or late puberty (52).

However, data on fracture risk during or after GnRHa treatment of transgender adolescents are lacking.

Because long-term (>1 year) hypogonadism may affect bone density, baseline BMD testing is indicated for transgender individuals prior to initiation of hormone therapy that lowers endogenous gonadal steroid levels for a significant period. Follow-up BMD testing in transgender should be done, when the results are likely to influence patient management, for example in individuals receiving PS (44). BMD measurement (using DXA) is recommended by Endocrine Society every 1–2 years during PS. Normal calcium intake and exercise may be beneficial in preserving bone health in transgender adolescents during PS (3).

## Effect of GnRHa on growth and height

Height SDS frequently decreases during the first 2 years of GnRHa treatment of transgender adolescents. Early experience with the Dutch protocol showed that height velocity (HV) of 21 adolescents with GD decelerated during triptorelin treatment for at least 2 years, especially in younger individuals (BA < 13 in girls, < 15 in boys), whose height SDS declined, while sitting-height:height ratio did not change (10). Schagen et al. (2016) observed that height SDS decreased significantly during the first two years of GnRHa treatment and did not change in the third year (12). Carmichael et al. found decreased height z-score with increased height during triptorelin monotherapy (started usually at stage 3-4) of 44 adolescents with GD (13).

TYCUS study of 55 transgender youth (52,7% AFAB) treated with GnRHa (84% histrelin, 16% leuprolide) for at least 10 months showed that initiation of GnRHa at early puberty resulted in growth rates similar with prepubertal controls, while beginning GnRHa later in puberty resulted in reduced HV. The majority (61,8%) of transgender adolescents started GnRHa at Tanner stage II. Median HV (5,1 cm/year) of transgender youth treated with GnRHa since stage II or III (n=50) for one year was not significantly different from HV of pre-pubertal age-matched cisgender controls. Starting GnRHa at stage IV was correlated with lower HV (1,6 cm/year). Age at GnRHa initiation was negatively associated with HV even when controlled for Tanner stage (53).

A recent retrospective cohort study of 146 AFAB treated with triptorelin before age 16 for at least 6 (mean 37) months showed that GnRHa does not have a significant negative effect on adult height, although it may raise it slightly above predicted, when started at a younger age. The cohort was subdivided into the pubertal (BA ≤ 14 before GnRHa initiation) and the postpubertal group (BA > 14). During GnRH, predicted adult height (PAH) increased by 2,4 cm, while mean height SDS decreased by 0,2. Height SDS of AFAB with BA > 12 (at GnRHa start) decreased more compared with those with BA ≤ 12 (whose height SDS was not reduced) during GnRHa, although height SDS at start of GAHT (after 3 years of GnRHa) was similar (between BA > 12 and ≤ 12). GnRHa also resulted in deceleration of bone maturation (increase in the difference between bone and chronological age), causing a delay of -1,9 years. Growth accelerated and height increased (by 5 cm) during subsequent testosterone treatment. Adult height SDS was comparable to height SDS at GnRHa start. Adult height in the pubertal group was higher than in the postpubertal (difference 3 cm) and younger BA (at GnRHa start) was associated with significantly greater adult height compared with PAH (1,2 cm/y), although the difference between adult and mid-parental height was not significant (in pubertal compared with postpubertal group) (54).

Another retrospective cohort study of 161 AMAB treated with triptorelin or pamorelin for 2,4 years (mean) and subsequently with estradiol (concurrently with GnRH until gonadectomy) showed that, although GnRHa and GAHT affect growth velocity (GV), their effect on adult height is not important. The cohort was divided into the pubertal group, with BA < 16 years (at GnRHa start), and the postpubertal group, with BA ≥ 16 and those who had completed growth clinically (without BA measurement). During GnRHa, PAH increased by 1,5 cm, while height SDS reduced continuously (–0,37/year). Average GV declined (by -1,9 cm) from 5,3 cm/year in the first year to 3,5 cm/year in the second year of (GnRHa) treatment. Bone maturation slowed down during GnRHa, which caused a delay in BA by 1,6 years, with more prolonged GnRHa management related to longer delay of BA (–0,5 years/year of GnRHa). During estrogen treatment, GV accelerated (height SDS increased). When GnRHa was followed by regular-dose (2 mg/d) 17β-estradiol, adult height was slightly lower than PAH at GnRHa initiation and close to target height. Growth reduction (adult height lower than PAH by 4,8) was achieved with high-dose (100-200 μg) ethinyl estradiol (EE). High-dose (6 mg/d) 17β-estradiol did not significantly reduce growth. Adult height was slightly below target height in AMAB treated with high-dose 17β-estradiol and EE. In the postpubertal group adult height was 2.7 cm lower than in the pubertal on regular-dose estradiol. The difference between adult and target height in 42 postpubertal AMAB was significantly larger compared to pubertal AMAB who received regular-dose estradiol (55).

Similarly, another retrospective study of 32 trans-adolescents showed that early PS (started at mean age 12,4-13) and GAHT do not affect final height. The difference between final and target height (for gender assigned at birth) was not statistically significant, which suggests that final height is closer to gender assigned at birth, rather than to experienced gender (56).

Overall, data at this point suggest that GnRH results in decrease of height SDS, with minimal effect on adult height. According to Endocrine Society Guidelines, “during treatment, adolescents should be monitored for negative effects of delaying puberty, including a halted growth spurt and impaired bone mineral accretion”. Measurement of height, weight, sitting height is recommended every 3–6 months during PS. Monitoring of BA by X-rays of the left hand can also help in growth evaluation (if clinically indicated) (3).

## Effect of GnRHa on body composition, body fat and weight

GnRHa may increase body weight and body fat (**Table 5**). Nokoff et al. observed higher percent body fat in transgender youth treated with GnRHa since early puberty (59). Van de Waal et al. noticed that fat mass percentage rose significantly during the first year of triptorelin treatment and stabilized afterwards, while lean body mass (LBM) fell significantly during the first year and did not change further (10).

**Table 5:** Effect of GnRHa on total body fat (TBF), gynoid/android fat, LMB and BMI.

	TBF		Gynoid fat		Android fat		LBM		BMI	
	AFAB	AMAB	AFAB	AMAB	AFAB	AMAB	AFAB	AMAB	AFAB	AMAB
Navabi B et al. 2021 (46)	↑ (+2,2%) TBF z-score ↔ (+0,13%)	↑ (+5,4%) TBF z-score ↑ (+1,05%)	↑ (+1,83%)	↑ (+7,17%)	↑ (+2,75%)	↔	↑ (+ 1,05 Kg), LBM z-score ↔	↔, LBM z-score↓ (–0,73)	↑ (+1,36 mean) (BMI z-score ↔)	↔ (+0,57 mean) (BMI z-score ↔)
Schagen S et al. 2016 (12)	↑ (+4,4% after 1 year of GnRHa)	↑ (+4,5% after 1 year of GnRHa)	ND	ND	ND	ND	↓ (-3,8% after 1 year of GnRHa)	↓ (-3,7% after 1 year of GnRHa)	↑ (+1,1 mean) after 1 year GnRHa, BMI SDS: ↑ by 0.17 (after 1 year of GnRHa), ↔ after 1^st^ year	↑ (+0,9 mean) after 1 year GnRHa, BMI SDS ↔ (after 1 year of GnRHa), ↑by 0.13 (at 2^nd^ year)
Klaver M et al. 2018 (57)	↑(+3% during GnRHa alone), ↓ (-3% at age 22 vs GnRHa start)	↑ (+6,6% during GnRHa alone, +10% at age 22 vs GnRHa start)	↑ (+3% during GnRHa alone), ↓ (-5% at age 22 vs GnRHa start)	↑ (+7% during GnRHa alone, +11% at age 22 vs GnRHa start)	↑ (+4% during GnRHa alone), ↔ (+1% at age 22 vs GnRHa start)	↑ (+5% during GnRHa alone, +9% at age 22 vs GnRHa start)	↓ (-3% during GnRHa alone), ↑ (+3% at age 22 vs GnRHa start)	↓ (-6% during GnRHa alone, -10% at age 22 vs GnRHa start)	↑ (+0,9 during GnRHa alone, +2,3 at age 22 vs GnRHa start)	↑ (+1,1 during GnRHa alone, +3 at age 22 vs GnRHa start)
Boogers LS et al. 2023 (58)	Z-scores ↑ + 0,47 during 3-year PS (+0,31 at 1 year)	z-scores↑ (+1,06) during 3 years of PS	ND	ND	Android/gynoid fat ratio↑ (+ 0,07) during 3- year PS	Android/gynoid fat ratio stable during 3- year PS	z-score ↓ (- 0,32) at 1 year PS (stable at 2^nd^-3^rd^ year)	z-score↓ (-1,13) during 3 years of PS	↑ + 2 kg/m^2^ (stable BMI SDS) during 3 years of PS	↑ + 1,8 kg/m^2^ (stable BMI SDS) during 3 years of PS

↔ no statistically significant change, ↑ increase, ↓ decrease; ND, no data.

Another retrospective study of 548 transgender adolescents (69% AFAB) showed that LBM z-scores decreased and fat mass z-scores increased gradually throughout 3 years of PS in AMAB and during the first year of PS in AFAB (and stabilized afterwards). Decline in LBM z-scores was greater after 3 years of triptorelin in late pubertal adolescents (58).

A retrospective study of 192 transgender adolescents (63% AFAB) treated with GnRHa showed changes of body composition and body shape toward the experienced sex at age 22. GnRHa was started at least at Tanner stage 2 (female) or 3 (male). In AMAB, a statistically significant enlargement in waist and hip circumference with reduction in WHR and a significant elevation in total body fat (TBF) and in body fat percentage in the android and gynoid region with a significant decline in LBM percentage were noticed. In AFAB, waist and hip circumference, WHR and LBM percentage increased significantly, while percentage of TBF and of gynoid fat decreased, without significant change of android fat. Alterations in body fat and LBM were not different at 22 years after adjustment for Tanner stage at GnRHa initiation, although beginning treatment at an earlier stage achieved a larger similarity of body shape to the experienced gender at 22 years in AFAB, a comparable tendency was observed in AMAB. SDS for WHR, body fat and LBM at age 22 in AMAB were more similar with ciswomen (than with AFAB), while in AFAB they were between reference values for ciswomen and cismen (57).

A retrospective cohort study of 192 transgender individuals (63% AFAB) showed that BMI and LDL increased during GnRHa monotherapy (started at Tanner stage 4 or 5 in most). BMI was also higher at age 22 (as compared with GAHT start), with higher prevalence of obesity in AMAB (9.9%) and AFAB (6.6%) than in controls (60).

Schagen et al. (2016) found that BMI SDS did not significantly change in the first year of triptorelin treatment and increased (by 0,13) in the second year in AMAB, while it increased in AFAB (by 0,17) in the first year and did not significantly change thereafter. LBM percentage significantly decreased during the first year of treatment, whereas fat percentage and absolute fat mass significantly increased (12).

In the recent study by Carmichael et al. treatment of 44 transgender adolescents with triptorelin caused no significant change in weight and BMI z-scores at 24 months, although a rise was observed at 36 months (13).

Joseph et al. observed a gradual rise in height and weight during 3-year GnRHa treatment of 31 adolescents with GD, with a larger upsurge in the height of AFAB and in the BMI of AMAB (49).

Navabi et al. (2021) found that BMI z-score did not change significantly after GnRHa, although BMI increased only in AFAB. TBF and gynoid fat increased significantly in all. In AFABs android (fat percentage) and LBM also rose significantly. LBM z-score decreased significantly and TBF (%) z-score increased in AMAB, while they did not change significantly in AFAB (46).

## Effect of GnRHa on blood pressure, lipids and glucose

GnRHa may increase blood pressure (BP), especially in AFAB. Diastolic BP percentiles increased significantly after GnRH treatment of 15 AFAB adolescents (at Tanner stage 4 or 5), although BP levels remained within the normal range for age and did not meet criteria for hypertension. Diastolic BP percentiles decreased to baseline after adding testosterone. Systolic BP did not change significantly (61). Klink et al. reported three cases of AFAB adolescents presenting arterial hypertension after triptorelin treatment. BP normalized after discontinuation of GnRHa in two of these cases, reoccurrence of hypertension after GnRHa restart was noticed in one case (62). Klaver et al. (2020) found that diastolic BP increased (by 4 mm Hg) in AMAB during GnRHa monotherapy (60). However, Nokoff et al. (2021) found lower (not statistically significant in AFAB, statistically significant in AMAB, in comparison with cisgender females and males, respectively) mean systolic BP among transgender adolescents on GnRHa (59). Stoffers et al. noticed non-significant increase in diastolic BP and decrease in systolic BP during GnRHa treatment of 62 transgender adolescents (50). Possible mechanism of hypertension during GnRH in AFAB is hypoestrogenism, as estrogen induces vasodilation (60). Therefore, BP monitoring is recommended before and every 3-6 months during GnRHa treatment by Endocrine Society (3).

Data on the effect of GnRHa on glucose metabolism are scarce. A small study of 17 transgender youth treated with GnRHa showed lower insulin sensitivity (higher HOMA-IR), more frequent dysglycemia (higher HbA1c), higher leptin and percent body fat than cisgender controls. In AFAB total (not percent) lean mass was lower (than matched cisgender females), percent lean mass was lower in AMAB. Insulin sensitivity (assessed by 1/fasting insulin) was inversely correlated with percent fat and with BMI percentile (59). However, Klaver et al. detected no significant change in glucose and HOMA-IR during GnRHa monotherapy of 192 transgender adolescents (60). Carbohydrate metabolism was not affected by GnRHa treatment of 21 adolescents in Amsterdam (10). No significant change in HbA1c was noticed after triptorelin treatment of trans-adolescents in the studies by Fisher and by Stoffers et al. (14, 50). In a multicenter analysis no statistically significant difference was found in the odds of dysglycemia in transgender youth receiving GnRHa monotherapy (63).

Limited evidence exists on the effect of GnRHa on lipids. Lipid metabolism did not differ after 2-year triptorelin treatment of 21 adolescents (10). Fisher found no statistically significant changes in lipid levels during triptorelin treatment, except a slight HDL increase in AMAB (14). Klaver et al. (2020) noticed that LDL increased (by 0,2 mmol/l) during GnRHa monotherapy, it increased further during combination GnRHa-GAHT treatment only in AFAB, while LDL was not significantly different at the age of 22 in transgender in comparison with cisgender peers (60). Stoffers et al. observed non-significant increases in total cholesterol (by 0,44 mmol/l), LDL (by 0,15 mmol/l), HDL (by 0,12 mmol/l) and triglycerides (by 0,01 mmol/l) during triptorelin treatment of 62 adolescents with GD (50). No association was found between GnRHa monotherapy of transgender youth and dyslipidemia in the recent analysis by Valentine et al. (63), although those treated with a combination of testosterone and GnRHa (or with combined oral contraceptive pills) had higher odds of dyslipidemia.

The latter multicenter retrospective, cross-sectional study analyzed cardiometabolic parameters in transgender and gender-diverse youth. GnRHa alone was not associated with significantly higher odds of cardiometabolic-related diagnoses. Norethindrone or medroxyprogesterone prescription for menstrual suppression in AFAB and antiandrogen spironolactone prescription for AMAB had no significant impact on cardiometabolic outcome, while AFAB receiving combined oral contraceptive pills had higher odds of overweight/obesity (63).

## Effect of GnRHa on fertility

Initiation of GnRH analogs during early puberty temporarily pauses germ cell maturation through suppression of gonadotropins and gonadal hormones.

Rise in both gonadotropins is the main drive for spermatogenesis initiation as well as for the pubertal proliferation of Sertoli cells, which provide the cytoarchitectural environment and the nutrients necessary for germ cell development. Spermatogenesis begins usually at Tanner stage 3 or (in around 20% of boys) at stage 2 (42). Therefore, at Tanner stage 2-3 some spermatogonia (possibly less spermatocytes as well) may be found in testes before starting PS in AMAB, while mature spermatozoa are absent.

Histological and immunohistochemical analyses of testicular tissue (obtained during orchiectomy) were carried out in a cohort study of 214 AMAB treated with triptorelin (since adolescence) or CA (in adulthood) in combination with estrogen (since age 16 or after 18) and orchiectomy (at the age of 18 or older, after at least one year on estrogen). Full spermatogenesis was noticed in 10 transwomen (4,7%), who had begun GnRHa at Tanner stage 4 or further. Immature germ cells (60-70% spermatogonia) were found in all transwomen who started GnRHa at Tanner stage 2 or 3 (64).

Suppression of testosterone halts spermatogenesis. In a prospective cohort study of 97 AMAB treated with CA plus estrogens, immunohistochemical staining of testicular tissue (obtained during gonadectomy) for 4 germ cell differentiation markers showed no spermatogenesis in 77,3% and partial spermatogenesis in 22,7% of the participants. In all AMAB with adequately suppressed serum testosterone levels (within female reference range), spermatogenesis was fully suppressed (65).

Oocytes go through meiotic maturation just before ovulation, which starts after menarche. Primary oocytes remain in meiotic arrest (at prophase I) until puberty. Positive feedback of estradiol on pituitary, which is necessary for midcycle LH surge and ovulation, occurs at mid-puberty before menarche. After preovulatory LH surge, meiosis I is resumed and enters metaphase I, during which conversion of primary to secondary oocyte takes place and formation of first polar body occurs (before ovulation). Meiotic maturation continues, until oocyte enters metaphase II. A second short meiotic arrest (at metaphase II) lasts until fertilization (42).

Consequently, PS before menarche and prevention of puberty completion in AFAB probably deters oocyte maturation, thus fertilization may be negatively affected. Therefore, for transgender men who received GnRHa since Tanner stages 2 or 3, there is probably no need for menstrual suppression or for contraception measures, as they have likely not achieved the maturity necessary for pregnancy, similar to congenital hypogonadotropic hypogonadism (33).

A mouse model, which mimicked AFAB youth receiving PS (followed by testosterone), showed absence of corpora lutea, suggestive of continued anovulation, at day 21 after GnRHa implantation, in parallel with suppressed HPG axis suppression and decreased ovarian and uterine weight. The number of primary follicles was significantly lower during GnRHa treatment compared with controls (66).

Delaying or temporarily discontinuing GnRHa to allow gamete maturation could be suggested, but it is usually not selected by transgender, due to development of secondary sex characteristics (3). Suppression of HPG axis by GnRHa is reversible and there is no published data that GnRHa harms ovarian function, ovulation or fertility permanently. Thus, sperm (or oocytes) may be collected at some time after GnRHa discontinuation (before GAHT initiation).

Studies in CPP children show only temporary reproductive effect of PS, which returns to normal after GnRHa discontinuation. In a small study of 7 boys with CPP treated with GnRHa for 5,6 years (mean), pubertal response of FSH/LH to GnRH test was noted within 1,5 years and spermarche 0,7 to 3 years after cessation of GnRHa (with normal semen analysis) (67). In a study of 87 girls with CPP ovarian volume decreased and uterine length remained unchanged during triptorelin, while they increased, with completion of ovarian and uterine development, appearance of menarche and rise of FSH and LH peaks (at LHRH test) about one year after the end of GnRHa treatment (68). In TAP-144-SR Japanese Study of 76 children with CPP treated with leuprorelin, menarche/remenarche appeared in more than 95% of girls at (mean) 17,5 months (with all idiopathic and half of organic CPP cases having ovulatory menstrual cycles) and serum testosterone raised to normal adult levels in all boys at 11 months (mean) after the end of GnRHa (69).

A prospective study of 15 girls with CPP (or early puberty) showed that AMH (a marker of ovarian reserve) decreased significantly (by 51%) after the first 3 months of leuprolide and remained partially suppressed after 12 months. AMH returned to pretreatment levels 6 months after stopping leuprolide, suggesting that GnRHa probably does not have a negative impact on future reproductive function (70).

However, no studies have examined the effect of GnRHa on long-term reproductive function of transgender adolescents and conclusions from studies of GnRHa use in CPP girls cannot be generalized to AFAB, because CPP girls eventually complete puberty, while AFAB may never complete it (as they usually start GAHT without discontinuing GnRHa).

Mature spermatozoa may be present at Tanner stages 3-4, thus sperm cryopreservation (before starting PS) is feasible in late puberty. According to de Nie et al., cryopreservation of mature spermatozoa (collected through TESE or during orchiectomy) may be feasible for 10% of AMAB starting GnRHa at Tanner stage 4-5, while it is not possible for those starting GnRHa at Tanner stage 2-3. Cryopreservation of testicular tissue (obtained during genital gender-affirming surgery) containing spermatogonial stem cells may be a future option for fertility maintenance for all transwomen starting GnRHa at Tanner stage 2-3 as well as for 90% of those starting GnRHa at Tanner stage 4-5 (64).

Another future experimental fertility option for transgender female may be uterus transplantation, which could improve dysphoric symptoms and offer the satisfaction of pregnancy and parenthood. However, its efficacy and safety in transgender women has not been proven and additional research is needed (71). There may also be legal barriers to its application.

Oocyte (or embryo) cryopreservation is feasible in late-pubertal perimenarchal AFAB. Oocyte retrieval requires ovarian stimulation by gonadotropins and transvaginal GD. Cryopreservation of ovarian tissue (usually of the cortex containing primordial follicles), which is obtained via biopsy or oophorectomy, is an option for fertility preservation, that could be useful in early-pubertal AFAB. *In vitro* growth of follicles (obtained from preserved ovarian tissue) is a promising method, which permits maturation of oocytes until metaphase II (71).

In conclusion, it is recommended that HCP inform transgender adolescents (before starting treatment) of the reproductive effects of gender-affirming treatments, including the potential loss of fertility (1). Endocrine Society recommends that clinicians “counsel all individuals seeking gender-affirming medical treatment regarding options for fertility preservation prior to initiating PS in adolescents” (3). Adolescents may not be competent enough to decide about fertility and may not fully realize the possible impact of gender-affirming treatments on fertility, thus consent and discussion should involve parent(s)/guardian(s) and the MHP.

## Effect of GnRHa on cognitive function

A study of 40 adolescents with GD (55% AFAB) showed that those receiving PS with GnRHa (n=20) did not develop brain functioning similar to the experienced gender, on the contrary sex differences in neural activation resembled their birth-assigned gender. GnRHa did not harm executive functioning, since it had no significant effect on ToL (Tower of London) performance (reaction times and accuracy) scores (an executive functioning task), although AMAB who received GnRHa had significantly lower accuracy scores. In AMAB who received GnRHa, a greater activation was found in functional MRI in some brain regions (in bilateral dorsolateral prefrontal cortex, left rostrolateral prefrontal cortex and left precuneus) during task load ToL performance (compared to female controls), while in AFAB treated with GnRHa bilateral precuneus activation was less (than male control) (72).

During normal puberty brain maturation occurs, which is associated with increases in sex steroids. For example, testosterone affects neural axon radial growth. There are concerns that suppression of sex hormones during puberty may prevent maturation of brain. A case report of a 11-year-old adolescent AMAB stated that brain white matter did not change during 28 months of GnRHa treatment and that operational memory decreased (by 9 points) after 22 months and remained stable after 28 months (of GnRHa) (73).

Expression of GnRH receptors has been found in the hippocampus, which regulates some cognitive functions, such as spatial orientation, learning and memory. Longtime peri-pubertal GnRHa treatment is related to alterations in mRNA expression of genes, involved in hormone signaling (GH, ESR1) and synaptic plasticity (VGF, NCAM1, LHX5) within the hippocampus of sheep (74). GnRHa does not significantly affect spatial orientation (74, 75). In a study using an ovine model, GnRHa did not affect maze traverse times of male sheep. Blockade of GnRH and testosterone affected the manner in which animals moved through a maze, increasing their emotional reactivity, which was restored with testosterone replacement. Blockade of GnRH signaling impaired long-term spatial reference memory, this effect was not restored with testosterone replacement (75).

## Other GnRHa adverse effects

In a prospective study adverse events were mild and frequent (in 22-25%) during the first two years of GnRHa treatment of adolescents with GD, while they were less common after 12 months (13). Hot flushes or mild headaches were the most common side effects (in 47,7% of participants during the first year). In two AFABs very small doses of ‘add-back’ estradiol decreased headaches and hot flushes. Mild fatigue was reported by 5–8% during the first two years, with no one reporting moderate or severe fatigue. Mild sleep problems, mood swings and weight gain were reported rarely (2-3%).

Similarly, hot flushes, mood swings, weight gain, and fatigue were the most common adverse effects of GnRHa, noted in 65% (11 out of 17) transgender adolescents, in a retrospective study (32). The first 11 AFAB treated (according to the Dutch protocol) with triptorelin (starting at pubertal stages 4-5) experienced numerous hot flashes, which occurred less often as months passes by (10).

Withdrawal bleeding may occur soon after GnRHa initiation (due to estrogen fall) before menses cease (12).

A few transgender adolescents have discontinued GnRHa due to side effects. In a small retrospective study of youth with GD in Canada, one (out of 27) transgender adolescent decided to cease GnRHa treatment because of emotional lability. In one AFAB leuprolide was changed to triptorelin due to sterile abscesses. One AFAB manifested leg pains and headaches during GnRHa, which receded. One young patient (with BMI above 85 percentile before treatment) gained 19 kg within 9 months of GnRHa (24).

In a retrospective study of 143 adolescents in the Netherlands, 3 subjects discontinued GnRHa due to possible side effects (mood disturbances/swings, suicidal thoughts, nausea/weight loss). One of them (AFAB) discontinued GnRHa after 4 months due to hot flushes, increase in migraine, fear of injections and stress (partly owing to school problems) and restarted GnRHa restart 5 months later (31).

## Discussion

PS can only be recommended to adolescents who meet the diagnostic criteria of GD (by DSM-5 and/or ICD-11) and have long-lasting intense GD, which aggravate with puberty onset. Possible mental issues should be addressed before treatment and informed consent (by the adolescent/parent) should be given, after counseling on possible reproductive effects of GnRHa.

The main aim of GnRHa treatment of adolescents with GD is the prevention of development of secondary sex characteristics (e.g. facial hair and voice deepening in AMAB), which aggravate distress. GnRHa can be administered only after the adolescents have entered Tanner stage 2. GnRHa are effective in arresting puberty and reversibly stop its progression to the following Tanner stages. GnRHa result in testicular volume reduction in AMAB, regression of breast development and menses cessation in AFAB. GnRHa may decrease the need for feminization/masculinization surgery (e.g. mastectomy in AFAB).

By alleviating the distress caused by puberty physical changes, GnRHa may improve psychosocial functioning, well-being, quality of life, emotional and behavioral (especially internalizing) problems and depressive symptoms, thus decreasing suicidality. However anxiety, body dissatisfaction and GD may not ameliorate.

PS can extend the diagnostic period and give transgender adolescents time to explore their gender identity before proceeding to GAHT and/or surgery. GnRHa administration may also reduce the necessary doses of GAHT.

However, PS may retard normal bone mass accrual. Most studies showed that BMD (and BMAD) z-scores (mainly in the LS) decrease during the first two years (rapidly during the first year) (49) of GnRHa treatment (and may stabilize during the third or fourth year) (48), especially in AFAB adolescents. Absolute BMD (and BMAD) values did not change significantly after GnRHa in the majority of the studies, although a reduction was noticed in a few studies (45, 48) particularly in late-pubertal AFAB. The decrease in z-scores is probably due to reduced bone turnover, as bone formation (mainly P1NP) and bone resorption (e.g. ICTP) markers decreased significantly during GnRHa, especially in younger adolescents (47, 48). Bone density increases during subsequent GAHT treatment, although z-scores may not reach levels before GnRHa start, especially in LS of AMAB (45, 47). However, most studies examined the effect of GnRHa initiated in late puberty on bone density. Evidence on the consequences of GnRHa started in early puberty on bone density in GD individuals (especially in AFAB) is relatively limited, although much bone has already developed in late puberty. Furthermore, inadequate data exist regarding the effect of GnRHa treatment for more than 2 years. CA may also result in decreased aBMD z-scores (mainly in LS), while lynestrenol probably does not affect normal bone development significantly (36). It is unknown whether GnRHa (or CA) increases the risk of fractures either in adolescence or later in life.

Height SDS decreases during the first two years of GnRHa treatment (10, 12, 54, 55), with HV lower than prepubertal controls, when GnRHa is started at Tanner stage 4 (53). Bone maturation may decelerate during GnRHa, which may cause a delay in BA by 1,6-1,9 years (54, 55). GnRHa does not seem to have a significant effect on adult height. However, adult height may be increased above predicted in AFAB, when GnRHa is initiated at a young BA. Adult height may be below predicted in AMAB, when GnRHa is followed by high-dose EE.

GnRHa, especially when started early at puberty, may cause changes in the body phenotype of transgender adolescents (mainly AFAB) resembling the desired sex (56). After GnRHa TBF, gynoid and android fat percentage increased, although Klaver et al. found that TBF and gynoid fat decreased (android fat increased non-significantly) in AFAB (57). LBM percentage (and LBM z-score) decreased in AMAB (12, 46, 57) (especially during the first year), while LBM increased in AFAB (12, 46) (without change in LBM z-score). Waist and hip circumference increase during GnRHa, with WHR increased in AFAB and decreased in trangirls (57). BMI z-score probably does not change significantly during GnRHa (46), although BMI (or BMI SDS) may increase during the first year of GnRHa treatment in AFAB (12, 46) and stabilize thereafter (12).

Through gonadotropin and gonadal steroid suppression, GnRHa initiation during early puberty reversibly halts normal pubertal gametes maturation, thus fertilization may be negatively affected. Therefore, transgender adolescents should be counselled about the possible reproductive effects of PS and about options for fertility preservation before starting treatment (1). Fertility preservation can be achieved via oocyte (or embyo) cryopreservation in late-pubertal AFAB and by sperm cryopreservation for late-pubertal AMAB.

GnRHa may cause hypertension, especially diastolic, mainly in AMAB adolescents, thus BP monitoring is recommended. The most common side effects of GnRHa are hot flushes, mood fluctuations, fatigue and headache. They are usually mild and rarely lead to GnRHa discontinuation.

A major drawback in examining the consequences of PS on transgender adolescents is the absence of large comparative studies. Limited evidence exists regarding long-term effect of PS on fertility, bone density and fracture risk, and neurocognitive development of transgender. There are no randomized controlled trials examining the efficacy and safety of GnRHa in adolescents with GD. The existing studies are limited in number, of small sample size, uncontrolled, observational, usually short-term, potentially subject to bias, so that evaluation of the evidence needs to be done with cautiousness. Moreover, conduct of the studies mainly in a limited number of tertiary referral centers and absence of research in low-income populations prevents extrapolation of their results. Gender-affirming treatment is more readily available in the western world, while in many less developed countries much more work is needed in these areas due to limited access to treatment and prejudice surrounding this topic.

## Conclusion

The inadequate published evidence from relatively small, uncontrolled studies suggests that PS (preferentially by GnRHa) can be beneficial in adolescents at Tanner stage 2 of further, who meet DSM-5/ICD-11 criteria of GD and give informed consent, after being counselled about potential reproductive effects, without having unaddressed mental health concerns. Advantages of PS are prevention of development of secondary sex characteristics and improvement of psychosocial functioning, especially decrease in emotional problems and depression. Disadvantages of GnRHa are the possible deceleration in bone mineral accrual and in height/growth velocity and the temporary pause in gametes maturation. Long-term randomized controlled studies with large study populations are needed in adolescents with GD to prove the positive and elucidate the possible negative effects of PS (on bone, growth, reproduction, cognition). Further research is needed to identify adolescents who might benefit, and those who are at greater risk.

## Author contributions

GB: Data curation, Methodology, Writing – review & editing. PG: Supervision, Writing – review & editing. SS: Writing – review & editing. PX: Conceptualization, Supervision, Validation, Writing – review & editing.
